# Supplementary material for: The uptake of population size estimation studies for key populations in guiding HIV responses on the African continent
Source: PLoS One. 2020 Feb 26;15(2):e0228634. doi: 10.1371/journal.pone.0228634 (PMC7043736; doi:10.1371/journal.pone.0228634)
Supplement: Attachment — Submitted filename: Response to Reviewers.docx [file pone.0228634.s001.docx]

Dear Dr. Beck and Reviewers,

Thank you for the review and constructive feedback of the manuscript (PONE-D-19-15539) submitted for consideration in the Project SOAR Collection. Please find below responses to feedback, and an outline of changes made to the manuscript for resubmission.

We have updated the manuscript to meet PLOS ONE’s style requirements following the style templates provided. We have also provided additional information on the methodology of the parent systematic review using the PRISMA Checklist. As part of this addressing point A, we have added more detail on the structure of the Global.HIV data repository as well as the data export and indicator selection process to clarify how PSE were identified in Phase 1 (Lines 178-201). To address point B, we have also added a description of the literature search and selection process of documents in Phase 2 (Lines 223-237).

**Responses to Reviewer #1 (R1):**

**R1 Comment: A sound review that I recommend for publication. It might be worth commenting more on the reasons for the findings such as PSE being often donor driven, and producing underestimations that make the validity of the results questionable and not very useful.**

Thank you for the recommendation and suggestion on adding commentary on reasons for findings. We agree that underestimations are a frequent observation that can lead to lack of utility and have noted this in the discussion (Line 462-463). While the motivations of PSE activity funders influencing utilization is an important consideration, evidence in documents conveying the reasoning of PSE funders/implementers was not examined in enough detail to be able to include this as a demonstrable factor influencing research utilization in the discussion section.

**Responses to Reviewer #2 (R2):**

**R2 Comment: In Line 200, the authored specified that the focus of the review was on PEPFAR and Global Fund investments. It may be beneficial to bring this fact to the forefront earlier (i.e. perhaps even in the abstract) and even include this in the limitations section.**

Thank you for this observation, and we have added a mention of this focus on PEPFAR and Global Fund investment in the abstract (Line 73) and as a limitation (Lines 456 – 459).

**R2 Comment: The article could benefit from a greater description of the gray literature sources used. There are many programs that conduct PSE for programming purposes but never publish the estimates in peer-reviewed journals. Were annual reports, programmatic study findings, etc. taken into account?**

Thank you for this for this important observation, and we have added a description of the gray literature sources used, as well as more detail on the eligibility criteria in the methods. In the Phase I results, we also have added a breakdown on the types of reports in grey literature to better describe their relationship with governmental and nongovernmental organizations (Lines 274-281). We broke down the types of grey literature reports identified, which include those with PSE activities as a component of HIV surveillance studies commissioned in collaboration with governmental organizations, PSE-specific studies, and NGO-led reports. **R2 Comment: Line 174-176: It was unclear which types of articles used in this review (i.e. gray and peer-reviewed).**

Thank you for this, and we have added more detail on the eligibility criteria to describe the sources included in the parent systematic review in the methods sections (Lines 175-177) and thus eligible for consideration in the Phase 1 PSE review. These sources include those available on the web from governmental on non-governmental sources (gray literature), as well as including sources published in a peer-reviewed journal or presented as an abstract at a scientific conference.

**R2 Comment: Line 267, 280, 287: Were the PSE used to "change" policy or just to inform it? Were there any instances where targets were not changed because estimates were as expected?**

Thanks for this important consideration and opportunity to convey how PSE references specifically relate to the ways program planning has been described. In most documents where we reported that PSE was used to change a policy or program, PSE rarely changed policy, but did change programs as described in the actions taken for planning of programs. About a third of documents that referenced PSE data, made no mention of using them to take actions in planning or any language commenting on the results of the estimates (Lines 378-381). But where action has been taken and attributed to the *existence* of PSE data, the document language specifies a different direction in planning regardless of the *results* of the estimates. We consider this to be a change in program planning rather than just informing it, and have observed this to be independent of whether or not the results of the estimates were as expected.
